# Supplementary material for: A Likelihood Approach for Real-Time Calibration of Stochastic Compartmental Epidemic Models
Source: PLoS Comput Biol. 2017 Jan 17;13(1):e1005257. doi: 10.1371/journal.pcbi.1005257 (PMC5240920; doi:10.1371/journal.pcbi.1005257)
Supplement: S1 File — (TAR.GZ) [file pcbi.1005257.s014.tar.gz › HSPH_Online-SI-Revision/PF08/n10-severe/n10-severe_PF2_table-8.pdf]

1	newly 3 weeks spec	291.	280.24	{193., 365.}	20.7766	0.0940522	newly 3 weeks cumul	664.	732.55	{524., 959.}	21.8208	0.0866598	newly 5 weeks spec	473.	336.73	{248., 458.}	30.6533	0.168323	newly 5 weeks cumul	1512.	1381.38	{982., 1793.}	19.8241	0.0937658	newly 59 weeks cumul	6569.	6027.84	{5212., 6901.}	10.65	0.0498763
2	newly 3 weeks spec	216.	242.46	{149., 331.}	28.3333	0.111627	newly 3 weeks cumul	425.	615.85	{409., 833.}	47.32	0.156102	newly 5 weeks spec	426.	319.72	{198., 446.}	30.1549	0.167327	newly 5 weeks cumul	1146.	1217.2	{782., 1646.}	24.6475	0.102127	newly 61 weeks cumul	6678.	6286.4	{5228., 7377.}	12.4064	0.0574101
3	newly 3 weeks spec	232.	255.45	{105., 489.}	51.7284	0.215234	newly 3 weeks cumul	460.	611.38	{276., 1138.}	56.7261	0.193108	newly 5 weeks spec	491.	355.6	{125., 689.}	46.2892	0.287485	newly 5 weeks cumul	1296.	1275.56	{519., 2386.}	47.7623	0.222674	newly 63 weeks cumul	5504.	6429.61	{4770., 8419.}	24.4017	0.0933426
4	newly 3 weeks spec	222.	235.46	{187., 287.}	15.3153	0.0623904	newly 3 weeks cumul	397.	635.36	{528., 753.}	60.0403	0.198426	newly 5 weeks spec	435.	273.88	{221., 335.}	37.4575	0.208999	newly 5 weeks cumul	1174.	1168.43	{962., 1380.}	13.0434	0.055998	newly 58 weeks cumul	6313.	5615.44	{5027., 6293.}	11.7047	0.0551817
5	newly 3 weeks spec	228.	271.73	{211., 338.}	21.9605	0.0806641	newly 3 weeks cumul	503.	725.68	{589., 895.}	44.2704	0.15193	newly 5 weeks spec	460.	319.58	{244., 400.}	32.1174	0.17436	newly 5 weeks cumul	1330.	1338.51	{1064., 1653.}	15.6699	0.0659165	newly 58 weeks cumul	6047.	5921.48	{5382., 6674.}	6.91384	0.0303869
6	newly 3 weeks spec	208.	244.01	{180., 322.}	24.6587	0.0909881	newly 3 weeks cumul	401.	639.75	{502., 806.}	59.5387	0.192255	newly 5 weeks spec	328.	298.99	{213., 399.}	20.5579	0.0975797	newly 5 weeks cumul	983.	1212.95	{918., 1539.}	26.587	0.0950959	newly 59 weeks cumul	5568.	6075.64	{5399., 6834.}	10.671	0.0432342
7	newly 3 weeks spec	360.	308.03	{206., 426.}	23.4806	0.118981	newly 3 weeks cumul	893.	821.93	{562., 1139.}	21.5308	0.102748	newly 5 weeks spec	397.	355.83	{234., 489.}	22.2594	0.10903	newly 5 weeks cumul	1675.	1511.82	{1045., 2063.}	21.6693	0.105333	newly 58 weeks cumul	4740.	6044.84	{5261., 6891.}	27.6557	0.103818
8	newly 3 weeks spec	247.	225.26	{134., 310.}	28.0891	0.135043	newly 3 weeks cumul	499.	557.52	{359., 743.}	28.7936	0.111597	newly 5 weeks spec	477.	306.44	{174., 410.}	40.5325	0.242946	newly 5 weeks cumul	1346.	1130.55	{686., 1478.}	27.2578	0.139576	newly 62 weeks cumul	5812.	6469.24	{5302., 7577.}	14.7897	0.0585294
9	newly 3 weeks spec	71.	271.57	{60., 573.}	288.972	0.463022	newly 3 weeks cumul	127.	590.2	{149., 1142.}	365.748	0.540975	newly 5 weeks spec	311.	430.3	{81., 975.}	87.6656	0.329895	newly 5 weeks cumul	564.	1380.27	{293., 2851.}	164.09	0.358723	newly 66 weeks cumul	6105.	6845.5	{3641., 8800.}	28.6775	0.122781
10	newly 3 weeks spec	214.	243.	{50., 534.}	76.6822	0.341864	newly 3 weeks cumul	335.	519.59	{127., 1100.}	94.3612	0.298034	newly 5 weeks spec	555.	392.56	{65., 879.}	61.4054	0.437138	newly 5 weeks cumul	1279.	1235.98	{262., 2651.}	66.5223	0.349159	newly 66 weeks cumul	6231.	6721.54	{3620., 8817.}	29.0528	0.128924
11	newly 3 weeks spec	107.	205.93	{59., 422.}	113.449	0.28265	newly 3 weeks cumul	156.	475.7	{158., 925.}	208.731	0.403286	newly 5 weeks spec	375.	308.05	{81., 698.}	61.3307	0.356822	newly 5 weeks cumul	750.	1045.41	{325., 2111.}	78.42	0.26529	newly 65 weeks cumul	5214.	5930.53	{3478., 8718.}	34.0558	0.138063
12	newly 3 weeks spec	89.	236.33	{93., 463.}	167.427	0.353752	newly 3 weeks cumul	220.	534.64	{230., 986.}	144.755	0.328503	newly 5 weeks spec	266.	383.02	{120., 771.}	78.9774	0.265238	newly 5 weeks cumul	648.	1225.71	{480., 2258.}	103.039	0.268362	newly 65 weeks cumul	5759.	7007.77	{4967., 8752.}	27.5487	0.104516
13	newly 3 weeks spec	161.	231.85	{129., 373.}	53.3354	0.167773	newly 3 weeks cumul	272.	568.6	{332., 862.}	109.044	0.289452	newly 5 weeks spec	444.	315.	{171., 466.}	38.2477	0.220602	newly 5 weeks cumul	1004.	1160.22	{639., 1740.}	36.3127	0.137152	newly 62 weeks cumul	6792.	6703.28	{5676., 7589.}	9.87603	0.0441111
14	newly 3 weeks spec	177.	228.37	{90., 441.}	61.0226	0.211876	newly 3 weeks cumul	299.	536.2	{229., 1042.}	88.602	0.231773	newly 5 weeks spec	540.	329.38	{125., 681.}	52.1593	0.347015	newly 5 weeks cumul	1231.	1146.79	{467., 2247.}	48.2916	0.230835	newly 64 weeks cumul	5655.	6630.44	{4886., 8387.}	22.7434	0.0878437
15	newly 3 weeks spec	125.	303.93	{12., 705.}	197.784	0.556327	newly 3 weeks cumul	202.	632.88	{36., 1357.}	255.198	0.53206	newly 5 weeks spec	451.	447.32	{21., 1192.}	86.7273	0.592805	newly 5 weeks cumul	842.	1463.32	{106., 3719.}	135.786	0.513999	newly 68 weeks cumul	6627.	6273.04	{1393., 8980.}	31.218	0.227311
16	newly 3 weeks spec	329.	250.33	{88., 444.}	45.8571	0.272352	newly 3 weeks cumul	674.	583.35	{247., 989.}	42.5534	0.222958	newly 5 weeks spec	678.	365.15	{131., 736.}	50.9071	0.377593	newly 5 weeks cumul	1835.	1254.2	{496., 2225.}	45.1989	0.288074	newly 64 weeks cumul	6828.	6676.88	{4700., 8585.}	16.9145	0.0783418
17	newly 3 weeks spec	187.	219.7	{104., 401.}	50.4813	0.187348	newly 3 weeks cumul	387.	524.17	{255., 846.}	52.9638	0.169681	newly 5 weeks spec	326.	326.34	{153., 670.}	51.5644	0.228681	newly 5 weeks cumul	968.	1129.69	{550., 2140.}	49.8151	0.185462	newly 64 weeks cumul	6543.	6370.84	{4404., 8377.}	19.1414	0.0878263
18	newly 3 weeks spec	201.	281.12	{147., 467.}	52.5672	0.169742	newly 3 weeks cumul	460.	688.54	{393., 1139.}	56.2957	0.173526	newly 5 weeks spec	415.	378.34	{174., 600.}	35.3012	0.17465	newly 5 weeks cumul	1164.	1406.01	{744., 2263.}	40.451	0.148744	newly 62 weeks cumul	6707.	6761.76	{5686., 7987.}	12.3829	0.0539918
19	newly 3 weeks spec	266.	260.22	{195., 322.}	16.594	0.0722621	newly 3 weeks cumul	588.	695.49	{548., 839.}	21.216	0.078164	newly 5 weeks spec	499.	300.34	{225., 384.}	40.3607	0.23237	newly 5 weeks cumul	1512.	1281.06	{1014., 1586.}	20.3214	0.0991102	newly 58 weeks cumul	6578.	5747.11	{5289., 6336.}	13.0032	0.0616357
20	newly 3 weeks spec	163.	266.37	{212., 325.}	63.4172	0.206164	newly 3 weeks cumul	399.	709.14	{582., 845.}	77.7293	0.244297	newly 5 weeks spec	300.	310.05	{251., 381.}	13.9767	0.0577934	newly 5 weeks cumul	931.	1307.9	{1072., 1568.}	40.4834	0.142009	newly 58 weeks cumul	5881.	5892.65	{5196., 6492.}	6.10253	0.0265742
21	newly 3 weeks spec	178.	304.12	{61., 719.}	117.449	0.357466	newly 3 weeks cumul	310.	637.22	{150., 1398.}	134.774	0.334665	newly 5 weeks spec	488.	476.52	{75., 1165.}	71.5902	0.403364	newly 5 weeks cumul	1116.	1526.41	{293., 3653.}	91.8342	0.352038	newly 66 weeks cumul	6367.	6875.43	{3229., 8870.}	29.4957	0.131719
22	newly 3 weeks spec	136.	294.87	{93., 533.}	129.596	0.310226	newly 3 weeks cumul	315.	668.81	{241., 1195.}	121.362	0.295262	newly 5 weeks spec	337.	440.98	{135., 859.}	72.7181	0.27577	newly 5 weeks cumul	870.	1484.72	{477., 2782.}	92.7816	0.266156	newly 64 weeks cumul	5004.	6991.24	{4838., 9017.}	42.1475	0.145246
23	newly 3 weeks spec	247.	227.82	{98., 395.}	40.8664	0.200817	newly 3 weeks cumul	543.	556.44	{255., 922.}	38.5304	0.169433	newly 5 weeks spec	429.	317.63	{134., 586.}	43.2238	0.254469	newly 5 weeks cumul	1291.	1149.14	{502., 1981.}	39.2579	0.198395	newly 63 weeks cumul	5813.	6468.09	{4907., 7997.}	19.4452	0.0786272
24	newly 3 weeks spec	98.	218.59	{97., 405.}	125.643	0.298813	newly 3 weeks cumul	155.	515.92	{252., 871.}	232.852	0.471284	newly 5 weeks spec	358.	319.56	{120., 592.}	46.1899	0.233923	newly 5 weeks cumul	700.	1104.77	{481., 2010.}	71.1014	0.20713	newly 64 weeks cumul	6876.	6686.38	{5013., 8299.}	14.9412	0.0685245
25	newly 3 weeks spec	16.	362.78	{66., 734.}	2167.38	1.16997	newly 3 weeks cumul	53.	730.74	{156., 1327.}	1278.75	0.986619	newly 5 weeks spec	207.	569.15	{90., 1306.}	196.217	0.417533	newly 5 weeks cumul	318.	1781.	{343., 3807.}	463.044	0.607318	newly 67 weeks cumul	5792.	7190.47	{4516., 9082.}	33.6756	0.13056
26	newly 3 weeks spec	172.	285.37	{54., 688.}	111.564	0.345418	newly 3 weeks cumul	282.	594.98	{137., 1300.}	136.475	0.329216	newly 5 weeks spec	397.	456.49	{71., 1099.}	79.8161	0.387422	newly 5 weeks cumul	956.	1435.71	{272., 3540.}	98.7019	0.343645	newly 66 weeks cumul	5226.	6938.21	{3228., 8949.}	45.7197	0.170834
27	newly 3 weeks spec	68.	424.6	{63., 858.}	530.735	0.618451	newly 3 weeks cumul	149.	824.35	{146., 1713.}	457.389	0.588801	newly 5 weeks spec	292.	648.39	{85., 1374.}	157.914	0.421076	newly 5 weeks cumul	586.	2039.36	{291., 4009.}	266.113	0.471698	newly 67 weeks cumul	6353.	7367.77	{4516., 9308.}	29.2657	0.119693
28	newly 3 weeks spec	54.	261.55	{51., 602.}	389.574	0.520652	newly 3 weeks cumul	104.	549.48	{129., 1126.}	429.885	0.574314	newly 5 weeks spec	285.	418.75	{67., 1022.}	102.474	0.381511	newly 5 weeks cumul	567.	1316.5	{254., 2985.}	160.642	0.366275	newly 66 weeks cumul	5472.	6943.74	{3426., 9143.}	39.9291	0.153401
29	newly 3 weeks spec	78.	291.73	{16., 705.}	311.423	0.582425	newly 3 weeks cumul	105.	567.5	{40., 1266.}	464.914	0.6403	newly 5 weeks spec	382.	499.58	{23., 1245.}	100.094	0.518711	newly 5 weeks cumul	647.	1481.86	{111., 3734.}	176.423	0.502842	newly 68 weeks cumul	5977.	6641.15	{2433., 8880.}	35.5655	0.191685
30	newly 3 weeks spec	42.	297.43	{16., 705.}	632.833	0.720697	newly 3 weeks cumul	59.	578.61	{40., 1383.}	895.78	0.807963	newly 5 weeks spec	274.	508.	{23., 1245.}	141.65	0.5216	newly 5 weeks cumul	436.	1509.58	{111., 3734.}	281.798	0.558972	newly 68 weeks cumul	6478.	6690.45	{2433., 8880.}	29.659	0.176653
31	newly 3 weeks spec	233.	260.63	{58., 572.}	71.97	0.326857	newly 3 weeks cumul	480.	580.38	{161., 1194.}	69.9958	0.285309	newly 5 weeks spec	432.	396.22	{81., 882.}	62.838	0.360457	newly 5 weeks cumul	1241.	1312.74	{311., 2833.}	66.2385	0.318492	newly 65 weeks cumul	5273.	6528.15	{3601., 8771.}	37.71	0.146055
32	newly 3 weeks spec	278.	303.6	{213., 412.}	21.2734	0.0838364	newly 3 weeks cumul	694.	807.11	{584., 1068.}	22.9467	0.085258	newly 5 weeks spec	361.	351.95	{253., 468.}	18.6343	0.0830631	newly 5 weeks cumul	1404.	1489.18	{1090., 1946.}	19.4231	0.0787618	newly 58 weeks cumul	4849.	6105.44	{5513., 6761.}	26.1712	0.0996631
33	newly 3 weeks spec	255.	249.78	{192., 319.}	15.2941	0.0675321	newly 3 weeks cumul	589.	672.42	{540., 858.}	17.6129	0.0666294	newly 5 weeks spec	409.	288.02	{222., 366.}	30.2885	0.163861	newly 5 weeks cumul	1337.	1233.08	{978., 1557.}	16.0464	0.074081	newly 58 weeks cumul	5959.	5780.08	{5298., 6357.}	6.11814	0.0272531
34	newly 3 weeks spec	330.	283.4	{212., 354.}	19.8848	0.0966088	newly 3 weeks cumul	818.	759.09	{592., 916.}	15.791	0.0722316	newly 5 weeks spec	455.	330.06	{250., 408.}	29.3319	0.155719	newly 5 weeks cumul	1672.	1401.03	{1078., 1710.}	20.6812	0.101698	newly 58 weeks cumul	6027.	6050.27	{5427., 6661.}	6.31608	0.0274412
35	newly 3 weeks spec	124.	279.09	{86., 543.}	137.815	0.316547	newly 3 weeks cumul	254.	619.95	{216., 1137.}	150.012	0.328499	newly 5 weeks spec	365.	423.4	{121., 870.}	60.5479	0.254978	newly 5 weeks cumul	843.	1397.77	{460., 2728.}	89.0593	0.259489	newly 65 weeks cumul	5774.	7177.05	{5400., 9012.}	27.8322	0.1034
36	newly 3 weeks spec	206.	225.22	{144., 315.}	27.8835	0.112108	newly 3 weeks cumul	457.	576.26	{381., 759.}	33.0372	0.116912	newly 5 weeks spec	363.	290.95	{175., 415.}	27.9477	0.149432	newly 5 weeks cumul	1101.	1127.12	{731., 1563.}	24.445	0.105189	newly 61 weeks cumul	6168.	6318.78	{5408., 7332.}	10.653	0.045204
37	newly 3 weeks spec	223.	219.39	{103., 408.}	43.4843	0.200538	newly 3 weeks cumul	420.	518.3	{257., 877.}	47.1571	0.172452	newly 5 weeks spec	351.	324.69	{134., 630.}	47.7407	0.236016	newly 5 weeks cumul	1055.	1111.77	{526., 2040.}	44.7687	0.192389	newly 64 weeks cumul	5466.	6620.91	{4930., 8402.}	25.7543	0.0964217
38	newly 3 weeks spec	171.	219.31	{100., 424.}	54.6374	0.186388	newly 3 weeks cumul	342.	514.02	{252., 872.}	62.9181	0.186882	newly 5 weeks spec	402.	322.33	{129., 696.}	50.4751	0.277253	newly 5 weeks cumul	991.	1106.56	{496., 2169.}	49.227	0.193839	newly 64 weeks cumul	6742.	6537.57	{4673., 8345.}	15.8406	0.0732375
39	newly 3 weeks spec	270.	269.09	{152., 377.}	25.4852	0.114754	newly 3 weeks cumul	603.	692.64	{436., 935.}	28.6169	0.11125	newly 5 weeks spec	373.	329.73	{191., 475.}	26.6836	0.134219	newly 5 weeks cumul	1298.	1325.72	{821., 1832.}	25.4222	0.110904	newly 60 weeks cumul	6183.	6182.56	{5130., 7135.}	10.6304	0.0471516
40	newly 3 weeks spec	213.	223.65	{151., 327.}	27.1784	0.10816	newly 3 weeks cumul	503.	585.16	{420., 837.}	26.5368	0.0948729	newly 5 weeks spec	419.	276.78	{181., 439.}	38.4726	0.221595	newly 5 weeks cumul	1236.	1116.11	{800., 1676.}	27.6076	0.127238	newly 60 weeks cumul	6409.	5942.88	{5092., 7168.}	12.2852	0.0568577
41	newly 3 weeks spec	69.	298.05	{16., 765.}	366.594	0.607962	newly 3 weeks cumul	109.	584.6	{40., 1398.}	461.339	0.634588	newly 5 weeks spec	305.	503.51	{23., 1245.}	125.23	0.515481	newly 5 weeks cumul	561.	1508.84	{111., 3798.}	211.561	0.519362	newly 68 weeks cumul	6138.	6660.2	{2433., 8880.}	33.8097	0.187821
42	newly 3 weeks spec	203.	244.2	{181., 313.}	24.7094	0.0918944	newly 3 weeks cumul	358.	635.3	{488., 767.}	77.4581	0.241816	newly 5 weeks spec	454.	298.	{217., 394.}	34.6256	0.193715	newly 5 weeks cumul	1061.	1205.59	{921., 1525.}	19.5203	0.0751133	newly 59 weeks cumul	6484.	6096.74	{5443., 6757.}	8.42443	0.0385445
43	newly 3 weeks spec	127.	359.89	{59., 794.}	206.512	0.408613	newly 3 weeks cumul	210.	715.58	{125., 1541.}	254.219	0.450511	newly 5 weeks spec	451.	590.91	{85., 1268.}	85.5011	0.36665	newly 5 weeks cumul	888.	1799.15	{307., 3850.}	135.425	0.364245	newly 67 weeks cumul	6305.	7263.44	{4564., 9323.}	29.0963	0.119606
44	newly 3 weeks spec	233.	211.85	{138., 311.}	28.1845	0.131167	newly 3 weeks cumul	482.	547.42	{378., 801.}	25.668	0.0950727	newly 5 weeks spec	403.	273.1	{176., 425.}	36.8238	0.211753	newly 5 weeks cumul	1184.	1064.34	{712., 1621.}	27.3108	0.128046	newly 61 weeks cumul	5552.	6104.15	{5219., 7177.}	14.1522	0.0569427
45	newly 3 weeks spec	215.	239.52	{100., 484.}	52.1674	0.212438	newly 3 weeks cumul	394.	558.12	{263., 1095.}	61.4213	0.195274	newly 5 weeks spec	444.	353.05	{137., 697.}	48.8716	0.276075	newly 5 weeks cumul	1183.	1207.01	{485., 2411.}	48.9425	0.217723	newly 64 weeks cumul	6681.	6717.97	{4818., 8664.}	15.9508	0.0707953
46	newly 3 weeks spec	191.	228.88	{101., 417.}	50.4921	0.1797	newly 3 weeks cumul	452.	559.13	{273., 978.}	46.9801	0.159603	newly 5 weeks spec	337.	313.64	{141., 653.}	48.2136	0.221974	newly 5 weeks cumul	1052.	1150.62	{536., 2235.}	45.5627	0.175917	newly 63 weeks cumul	5089.	6345.	{4792., 7792.}	27.9391	0.103017
47	newly 3 weeks spec	210.	256.1	{80., 439.}	62.5619	0.24969	newly 3 weeks cumul	472.	585.87	{217., 992.}	58.0784	0.222688	newly 5 weeks spec	303.	387.94	{119., 756.}	68.495	0.262213	newly 5 weeks cumul	1066.	1294.78	{434., 2210.}	60.803	0.241821	newly 64 weeks cumul	5261.	6959.08	{4839., 8925.}	35.0675	0.125922
48	newly 3 weeks spec	153.	223.94	{151., 333.}	48.5621	0.154777	newly 3 weeks cumul	281.	566.27	{386., 826.}	101.52	0.285543	newly 5 weeks spec	442.	289.97	{181., 420.}	37.1154	0.216669	newly 5 weeks cumul	972.	1111.95	{731., 1630.}	28.6512	0.108166	newly 61 weeks cumul	6845.	6353.32	{5382., 7406.}	11.4355	0.0534385
49	newly 3 weeks spec	191.	208.27	{97., 373.}	43.7539	0.179385	newly 3 weeks cumul	421.	496.03	{263., 799.}	42.0214	0.157487	newly 5 weeks spec	403.	301.3	{136., 566.}	44.4169	0.252547	newly 5 weeks cumul	1126.	1053.92	{511., 1882.}	39.9094	0.187731	newly 63 weeks cumul	5314.	6570.79	{4614., 8027.}	28.3788	0.106844
50	newly 3 weeks spec	165.	300.47	{17., 765.}	143.012	0.517004	newly 3 weeks cumul	248.	629.79	{41., 1398.}	198.415	0.492873	newly 5 weeks spec	521.	477.86	{29., 1245.}	78.9981	0.55209	newly 5 weeks cumul	1082.	1504.72	{116., 3798.}	105.445	0.484407	newly 68 weeks cumul	6795.	6493.17	{2360., 8979.}	27.6946	0.180172
